# Osteoporosis development and vertebral fractures after abdominal irradiation in patients with gastric cancer

**DOI:** 10.1186/s12885-018-4899-z

**Published:** 2018-10-11

**Authors:** Gokhan Yaprak, Cengiz Gemici, Sule Temizkan, Sevim Ozdemir, Berfu Cinkit Dogan, Ozgur Ozan Seseogullari

**Affiliations:** 1 Department of Radiation Oncology, Dr. Lutfi Kirdar Kartal Education and Research Hospital, Istanbul, Turkey; 2 Department Endocrinology, Dr. Lutfi Kirdar Kartal Education and Research Hospital, Istanbul, Turkey; 3 Department of Family Physician, Dr. Lutfi Kirdar Education and Research Hospital, Istanbul, Turkey; 4 Department of Radiation Oncology, Biruni University Medicana International Hospital, Istanbul, Turkey

**Keywords:** Gastric cancer, Bone mineral density, Osteoporosis, Insufficiency fracture, Abdominal radiotherapy

## Abstract

**Background:**

Decrease in bone mineral density, osteoporosis development, bone toxicity and resulting insufficiency fractures as late effect of radiotherapy are not well known. Osteoporosis development related to radiotherapy has not been investigated properly and insufficiency fractures are rarely reported for vertebral bones.

**Methods:**

Ninety-seven patients with gastric adenocarcinoma were evaluated for adjuvant treatment after surgery. While 73 out of 97 patients treated with adjuvant chemoradiotherapy comprised the study group, 24 out of 97 patients with early stage disease without need of adjuvant treatment comprised the control group. Bone mineral densities (BMD) of lumbar spine and femoral neck were measured by dual energy x-ray absorptiometry after surgery, and one year later in both groups.

**Results:**

There was statistically significant decline in BMDs after one year in each group itself, however the decline in BMDs of the patients in the irradiated group was more pronounced when compared with the patients in the control group; *p* values were 0.02 for the decline in BMDs of lumbar spine, and 0.01 for femoral neck respectively. Insufficiency fractures were observed only in the irradiated patients (7 out of 73 patients) with a cumulative incidence of 9.6%.

**Conclusions:**

Abdominal irradiation as in the adjuvant treatment of gastric cancer results in decrease in BMD and osteoporosis. Insufficiency fracture risk in the radiation exposed vertabral bones is increased. Calcium and vitamin D replacement and other measures for prevention of osteoporosis and insufficiency fractures should be considered after abdominal irradiation.

## Background

Radiation related late toxicity is a major concern and is well defined for certain tissues and organs in radiation oncology practice [1]. Altered bone metabolism and bone toxicity due to radiation has not been studied extensively and it is not well known. Bone toxicity as a late effect of radiotherapy is multifactorial and results from direct and indirect effects of irradiation on bone [2, 3]. Bone fractures are the worst adverse effects of radiation on bone tissue and these fractures are generally called as insufficiency or stress fractures (IFs), which are generally the subgroup of fractures that result from normal or physiologic stress applied to weakened bone.

Cancer survivors are at increased risk of osteoporosis and subsequent fractures related to the type of cancer and its treatment. While decreased bone mineral density (BMD) and osteoporosis is a well known complication of certain cancer treatments such as endocrine therapies and chemotherapy, its association with radiotherapy has not been reported and relation between osteoporosis and radiation has not been investigated sufficiently [4, 5]. Decrease in BMD and osteoporosis seen after radiotherapy is often an indirect effect of irradiation and results either from pelvic irradiation induced ovarian or testicular failure or metabolic syndromes which develop after cranial irradiation of hypopyhseal pituitary stalk in survivors of childhood tumors [6–9]. Osteoporosis is a common complication of additional treatments during radiotherapy such as endocrine manipulations in breast and prostate cancer. These additional treatments during or after radiotherapy increase the risk of decline in BMD and increase radiation associated fracture risk [3, 10, 11].

Radiation induced decrease in BMD, osteoporosis and insufficiency fractures have been rarely reported prospectively and are mostly restricted to patients who were treated with pelvic irradiation [2, 3, 9, 12–15]. The reported cumulative incidences of IFs after pelvic radiotherapy are indeed very high and range between 8.2 to 45.2% in cervical cancer, 9% to 11.2% in rectal cancer, and 6.8% in prostate cancer [2, 3, 9, 12–15]. Although osteoporosis and IFs are observed frequently after pelvic irradiations, bone toxicity related to radiotherapy is a neglected and unknown toxicity among radiation oncologists.

Abdominal irradiation as in the adjuvant treatment of patients with operated gastric cancer may result in decline in BMD and osteoporosis. This is an indirect effect which results from malabsorption of calcium and vitamin D and other micronutrients related with bone metabolism due to radiation induced late tissue toxicity on remnant stomach, small intestine and pancreatic tissue [16–18]. Beside this indirect effect of abdominal irradiation on bone tissue, there is also a direct bone toxicity. Vertebral bones are exposed to irradiation during adjuvant abdominal radiotherapy.

In this prospective nonrandomized study we investigated BMD changes, osteoporosis risk, and insufficiency fracture incidence in patients who were treated with adjuvant abdominal irradiation for curatively resected gastric cancer.

## Methods

The eligibility criteria included patients with histologically confirmed adenocarcinoma of the stomach after curative surgical resection. These patients were evaluated for adjuvant treatment after surgery. Patients between the ages of 18 and 70 were included in the present study. While seventy-three patients presenting with serosal or adjacent visceral organ invasion (pT3, T4), or with involved lymph nodes were considered suitable for adjuvant treatment and comprised the study group, twenty-four patients who did not need adjuvant treatment (pT1, pT2 and pN0) comprised the control group. No patients with chronic diseases such as hyperthyroidism, liver cirrhosis and renal failure were included in the study. Patients were also excluded if they used drugs known to influence bone metabolism such as biphosphonates, corticosteroids, estrogens. Patient characteristics comprising T and N stages, surgical resection type were summarized in Table 1.

**Table 1:** Patient charactheristics

	Study group Radiotherapy (+)	Control group Radiotherapy (−)
T Stage		
T1	3 (4%)	11 (46%)
T2	11 (15%)	13 (54%)
T3	11 (15%)	–
T4	48 (66%)	–
N Stage		
N0	15 (21%)	24 (100%)
N1	28 (38%)	–
N2	16 (22%)	–
N3	14 (19%)	–
Surgical procedure		
Subtotal	41 (56%)	13 (54%)
Total	32 (44%)	11 (46%)

Adjuvant treatment plan in the study group was similar to the intergroup 0116 trial presented in 2001 by MacDonald et al. [19]. Patients received either bolus or infusional 5-fluorouracil, one cycle before, two cycles concomitant with, and one cycle after radiation treatment. Radiation was delivered with either 6 or 15 MV photons by three-dimensional conformal technique including anastomosis, tumor bed and regional lymphatics similar to the technique described by Smalley et al. [20]. The radiation dose prescribed was 46 Gy in 23 fractions with 2 Gy fractions per day, 5 days per week for 5 weeks. The study and the study protocol were reviewed and approved by the Ethics committee of the Dr. Lutfi Kirdar Kartal Education and Research Hospital (67–2015). Written informed consent were obtained from all the patients in the study.

BMD of lumbar spine and femoral neck were obtained prospectively by dual energy x-ray absorptiometry (DEXA, GE Prodigy; Lunar Radiation, Madison, WI, USA). The results of BMD were expressed as absolute values (g/cm^2^). T scores and Z scores were provided by the GE-Lunar database. BMDs were obtained after operation for all the patients, and one year later after operation for patients in the control group, and one year after the end of radiotherapy for patients in the study group. One year timing was considered enough for late radiation toxicity evaluation and osteoporosis development. All the patients were followed up regularly and any incidence of insufficiency fractures was recorded for both groups. Minimum, maximum and mean radiation doses were recorded in the study patients for the fractured vertebras.

Statistical analysis was performed with the statistical package for the social sciences (SPSS 17.0). Within group significance was determined by the Wilcoxon signed rank test and between group differences by the Mann-Whitney and x^2^ tests. A *p* value of less than 0.05 was considered statistically significant. Results were expressed as mean ± SD.

## Results

Demographic features of the study and control groups are summarized in Table 2. At baseline there was no difference in age, sex, gastric resection type (total or subtotal gastrectomy), body mass index (BMI), BMD of lumbar spine and femoral neck between two groups.

**Table 2:** Initial comparison of study and control group patients

	Study group Radiotherapy (+) N:73	Control group Radiotherapy (−) N:24	p
Age (years)	54.84 ± 12.9	55.92 ± 11.6	0.85
Gender(F/M)	18/55	9/15	0.22
BMI (kg/m^2^)	23.52 ± 3,85	24.8 ± 4.33	0.29
BMD (g/cm^2^) Lomber spine	1078 ± 164	1152 ± 232	0.09
BMD (g/cm^2^) Femoral neck	918 ± 155	987 ± 203	0.08

*BMI* Body mass index, *BMD* Bone mineral density (g/cm^2^)

One year after adjuvant radiotherapy in the study group and one year after surgical resection in the control group, there was statistically significant decline in BMDs in each group itself; *p* values were < 0.001 for BMDs of both lumbar vertebra and femoral neck in the study group, and 0.007, and 0.001 for lumbar vertebra and femoral neck in the control group. However the decline in BMDs of the patients in the study group was more pronounced when compared with the patients in the control group; p values were 0.02, and 0.01 for BMDs of lumbar vertebra and femoral neck respectively. Table 3 summarizes BMD decreases in patients during the study period in both groups and in between the two groups themselves.

**Table 3:** Comparison of BMIs and BMDs during the study period in both groups and in between the two groups themselves

	Study group Radiotherapy (+)	Control group Radiotherapy (−)	p
BMI (kg/m^2^) - initial	23.52 ± 3.85	24.8 ± 4.33	0.29
BMI (kg/m^2^) - 1st year	22.1 ± 3.21	25.1 ± 4.74	0.005
P	0.001	0.33	
BMD Lomber Spine - initial	1078 ± 164	1152 ± 232	0.09
BMD Lomber Spine - 1st year	975 ± 208	1093 ± 211	0.02
p	0.001	0.007	
BMD Femoral Neck - initial	918 ± 155	987 ± 203	0.08
BMD Femoral Neck - 1st year	832 ± 166	935 ± 198	0.01
P	0.001	0.001	

*BMI* Body mass index, *BMD* Bone mineral density (g/cm^2^)

Insufficiency fractures were observed only in the irradiated patients (7 out of 73 patients) after a mean follow-up of 36 months with a cumulative incidence of 9.6%. The mean time for the development of IFs was 22 months (range, 12–36 months). IFs were all observed in the radiation exposed vertebral bones, and not outside the radiation field. The vertebral fractures were in the 12th thoracal vertebra (2 out of 7 patients), 2nd lumbar vertebra (2 out of 7 patients), and 3rd lumbar vertebra (3 out of 7 patients). IFs were detected by back pain which was neuropathic and radicular in character. These vertebral fractures were often considered as bone metastases of the original tumor and referred for palliative radiotherapy by consulting medical oncologists. Pathologic confirmation was possible in 2 out of 7 patients who underwent vertebroplasty. For other patients malignancy was excluded due to radiologic and clinical characteristics of the fractured vertebrae: only one vertebral involvement with no other bony lesion, structural integrity of posterior parts of the involved vertebrae, no spinal instability, and no diffusion restriction in magnetic resonance imaging. Table 4 summarizes the clinical information for the patients with vertebral fractures.

**Table 4:** The clinical information for the patients with vertebral fractures

Patients	1st	2nd	3rd	4th	5th	6th	7th
Age	42	63	59	38	69	70	65
Gender	Male	Female	Male	Female	Female	Male	Male
Tumor Location	Cardia	Antrum	Corpus	Cardia	Antrum	Pylor	Antrum
Fractured Vertebrae	T12	L3	L2	T12	L3	L3	L2
Latency Period (Months)	26	17	23	36	12	24	16
Minimum Radiation Dose (cGy)	2672	1556	2086	2484	1094	1627	1874
Mean Radiation Dose (cGy)	3930	3203	3635	3812	2985	3124	3525
Maximum Radiation Dose (cGy)	4755	4689	4735	4732	4620	4605	4710

Pain relief was achieved by nonsteroidal anti-inflammatory drugs, vertebroplasty or stabilisations by corset. All of the symptoms were successfully managed with conservative treatment and patients were relieved from unnecessary irradiations and chemotherapy.

## Discussion

Decrease in bone mineral density, osteoporosis development and distruption of bone microarchitecture leading to compromised bone strength and increased fracture risk is very common and a major public health problem. Osteoporosis is the most common bone disease in humans and is a risk factor for fracture [21]. Fractures and their complications are clinical sequelae of osteoporosis. Radiotherapy may lead to osteoporosis by direct and indirect mechanismes and IFs develop frequently after pelvic irradiations in clinical practice [2, 3, 9, 12–15]. Unfortunately many oncologists are not aware of these fractures, and sometimes these fractures may be misinterpreted as bone metastases resulting in unnecessary interventions and even medicolegal problems.

Since 2001, after the pivotal intergroup 0116 trial, abdominal radiotherapy together with 5FU based chemotherapy was established as the standart of care in patients with serosal or adjacent visceral organ invasion and or lymph node involvement after curative resection of gastric cancer [19]. Many patients are exposed to abdominal irradiation and its related and some still unknown complications (exocrine, endocrine, renal, e.t.c) [16–18]. Osteoporosis and IFs are other possible and unknown complications that may develop after abdominal irradiation.

Gastrectomy itself is a known risk factor for decreased bone mass, osteoporosis and associated fractures [22–24]. The prevalence of osteoporosis is very high in patients who are operated by total or subtotal gastric resection either for gastric cancer or for peptic ulcer disease [23]. American Gastroenterological Association recommends dual-energy X-ray absorptiometry (DEXA) evaluation in patients who underwent gastric resection [25]. Subtotal and total gastrectomy alters normal gastrointestinal physiology. Poor absorption of vitamin D and calcium results in secondary hyperparathyroidism which in turn results in osteomalacia and osteoporosis [23]. Impaired protein nutrition secondary to reduced intake and absorption problems after gastrectomy alters protein metabolism and thus collagen matrix formation [26–28]. In our study we observed a decrease in BMDs and osteoporosis development in both study and control groups after one year of follow-up. The decrease in BMDs of lumbar vertebra and femoral neck region was statistically significant in each group itself. It may be suggested that these findings are mainly due to gastric resection and associated malabsorption in the patients. However when we compare the BMD values in the study group with the control group at the end of study, we see that this decrease was more marked in patients who underwent radiotherapy after gastrectomy compared to patients who only underwent gastrectomy, and *p* values were 0.02 and 0.01 for lumbar vertebra and femoral neck region respectively. Age and sex were not determinants of the difference in the decrease in BMDs between the two groups since these variables were well balanced and not statistically different between the two groups.

Malabsorption and malnutrition due to gastrectomy is aggravated with the addition of adjuvant abdominal radiotherapy after surgical resection [16–18]. Malabsorption and malnutrition is responsible for the decline in BMD and osteoporosis development in this prospective study. Although patients in the study and control groups had similar body mass indexes (BMI) at the onset of the study (*p* = 0.29), BMI had significantly fallen after one year in the study group compared to the patients in the control group, *p* values were 0.001 and 0.33 in study and control groups at the end of the study (Table 3). This is an indirect indicator of malabsorption and malnutrition in the irradiated patients. Abdominal irradiation made the nutritional status worse in the study group when compared with control group who underwent solely gastrectomy (*p* = 0.005).

Decrease in BMD, osteoporosis and IF development related to radiotherapy are not studied extensively and many radiation oncologists are not aware of radiation related bone complications. Insufficiency fractures are increasing nowadays with the implementation of stereotactic irraditions but the mechanism in this setting is probably due to direct bone toxicity of high dose irradiation rather than a decrease in BMD and osteoporosis development [29]. Beside a decrease in BMD and osteoporosis development as an indirect effect of abdominal irradiation on bone metabolism, there is also a direct effect of abdominal irradiation on vertebral bones. All the fractures in the study group were observed within the radiation field but not outside as observed in the pelvic IFs developed after pelvic irradiations. Radiation damages and occludes microvasculature of mature bone and causes stasis of osteoclasts and osteoblasts [3, 30–33]. Irradiation reduces osteoblast number, arrests osteoblast cell cycle progression, increases susceptibility to apoptosis, implying that reduced bone formation is a major contributor to radiotherapy induced bone damage [3, 30–33]. Radiation damages also bone matrix, increases marrow adiposity and decreases vascular supply to the bone [3, 30–33]. Why IFs are observed within the radiation field rather than outside is due to this direct effect of irradiation on bone besides indirect effect of irradiation on bone metabolism.

There are certain characteristics of IFs differentiating them from bone metastases. As stated above they almost always develop within the previous radiation field and most of the time they present with single bone involvement. Clinical and radiologic details which are mentioned in the results section differentiate IFs from bone metastases. These patients are most of the time osteoporotic and they have other associated conditions that already predispose them to osteoporosis like the hormonal treatment of breast or prostate cancer. This prospective study has shed light on both direct and indirect effects of radiation on bone metabolism, osteoporosis development and resulting insufficiency fractures.

Decrease in BMD, development of osteoporosis and IF risk related to abdominal irradiation has not been investigated prospectively before. Osteoporosis is observed in patients after abdominal irradiation and IF risk is a significant late effect of radiotherapy in these patients. As clinicans, we must be aware of this possible complication and take two points into consideration. First of all we should not think of vertebral fractures as the metastases of primary tumor especially if the involved bone is already in the irradiated area. Secondly we should follow BMDs in this group of patients with DEXA and take preventive measures against development or progression of osteoporosis and consult these patients with endocrinologists.

When we evaluated the seven patients with IFs (Table 4), we realized that the fractured vertebras were exposed to high radiation doses. This is due to three-dimensional conformal radiotherapy planning with no attention to the radiation doses received by the vertebral bones. The mean radiation doses were around or above 30 Gy in the fractured vertebrae. We should try to decrease mean radiation doses for the vertebrale bones within the radiation field especially in the elderly and already osteoporotic patients. It is better to offer intensity modulated radiotherapy to these patients in order to prevent future fracture risk. Biphosphonates together with calcium and vitamin D are also very important in preventing osteoporosis and lowering the fracture rates. They have been shown to be effective in preventing skelatal related adverse events and should be considered in these patients [34, 35].

## Conclusions

Athough bone toxicity is a frequent complication of radiotherapy in cancer survivors, radiation induced osteoporosis and insufficiency fractures as late effects of radiotherapy are rarely recognized by radiation oncologists. Insufficiency fractures as the worst outcome of radiation induced bone toxicity result in significant morbidity and financial burden. It is very important to keep in mind this possible and in fact common complication especially in old and postmenopausal patients receiving hormonal manipulations resulting in osteoporosis.

## Abbreviations

BMD: Bone mineral density
BMI: Body mass index
DEXA: Dual energy x-ray absorptiometry
IFs: Insufficiency fractures
SPSS: Statistical package for the social sciences
